# Enhancing durability and sustainable preservation of Egyptian stone monuments using metabolites produced by *Streptomyces exfoliatus*

**DOI:** 10.1038/s41598-023-36542-1

**Published:** 2023-06-10

**Authors:** Basma T. Abd-Elhalim, Bahaa A. Hemdan, Salwa M. El-Sayed, Mahgoub A. Ahmed, Sodaf A. Maan, Samah H. Abu-Hussien

**Affiliations:** 1 Department of Microbiology, Faculty of Agriculture, Ain Shams University, Cairo, 11566 Egypt; 2 Water Pollution Research Department, Environmental Research and Climate Change Institute, National Research Centre, 33 El-Bohouth St., Dokki, 12622 Giza Egypt; 3 Department of Biochemistry, Faculty of Agriculture, Ain Shams University, Cairo, 11566 Egypt; 4 Department of Conservation, Faculty of Archaeology, South Valley University, Qena, Egypt

**Keywords:** Industrial microbiology, Biochemistry, Biotechnology, Immunology

## Abstract

Despite their threatens for Egyptian stone monuments, A few studies focused on using biocontrol agents against deteriorative fungi and bacteria instead of using chemical assays that leave residuals leading to human toxicity and environmental pollution. This work aims to isolate and identify fungal and bacterial isolates that showed deteriorative activities from stone monuments in Temple of Hathor, Luxor, Egypt, as well as determine the inhibitory activity of metabolites produced by *Streptomyces exfoliatus* SAMAH 2021 against the identified deteriorative fungal and bacterial strains. Moreover, studying the spectral analysis, toxicological assessment of metabolites produced by *S. exfoliatus* SAMAH 2021 against health human cell fibroblast, and colorimetric measurements on the selected stone monuments. Ten samples were collected from Temple of Hathor, Luxor, Egypt. Three fungal isolates and one bacterial isolate were obtained and identified as *A. niger* isolate Hathor 2, *C. fioriniae* strain Hathor 3, *P. chrysogenum* strain HATHOR 1, and *L. sphaericus* strain Hathor 4, respectively. Inhibitory potential of the metabolites in all concentrations used (100–25%) against the recommended antibiotics (Tetracycline 10 µg/ml and Doxycycline (30 µg/ml) showed an inhibitory effect toward all tested deteriorative pathogens with a minimum inhibition concentration (MIC) of 25%. Cytotoxicity test confirmed that microbial filtrate as the antimicrobial agent was safe for healthy human skin fibroblast with IC_50_ of < 100% and cell viability of 97%. Gas chromatography analysis recorded the existence of thirteen antimicrobial agents, Cis-vaccenic acid; 1,2-Benzenedicarboxylic acid; ç-Butyl-ç-butyrolactone and other compounds. Colorimetric measurements confirmed no color or surface change for the limestone-treated pieces. The use of the metabolite of microbial species antimicrobial as a biocontrol agent raises contemporary issues concerning the bio-protection of the Egyptian monuments to reduce chemical formulas that are toxic to humans and pollute the environment. Such serious problems need further investigation for all kinds of monuments.

## Introduction

Conserving monuments from deterioration is indispensable, especially for archaeologists. Since ancient antiques such as stone monuments may be infected by harmful microbes, like humans, this worsens their quality^1,2^. The Egyptian monuments have always been in danger. They have never been adequately valued, preserved, or marketed. As Archaeology has excellent value in human civilization and is a witness to the history of ancient man, and represents a tremendous mortal leap, we should control the evolution of the deteriorative fungal and bacterial species for them, especially the ancient Egyptian stones. Since Egypt has been a country of civilization and antiquities for thousands of years, this study is of particular importance indeed^3^. Many efforts have been increased to protect Egyptian stone monuments against fungal and bacterial biodeterioration to reduce chemicals using integrated novel management strategies providing environmental and economically feasible alternatives. As is sufficiently known, improper and uncontrolled chemical fungicides have caused severe side effects, such as residual poisoning and ecological contamination, necessitating an urgent shift to biological control of some commercial antifungal treatments^4,5^.

*Actinomycetes*, particularly the *Streptomyces* species, became well recognized not only for degrading organic substances such as lignocellulosic biomass, starch, and chitin in sediment^6^, but also for assembling a variety of antimicrobial peptides as alternative bioactive molecules for commercial antibiotics^7^. Further, the observation that these microbial species owned substantial fungal and bacterial suppressive possibilities, despite the reality that the function of *Streptomyces* species in safeguarding and maintaining stone monuments, has attracted little investigation, clearly demonstrating their significance in Egypt's monument preservation^8^.

Environment-related factors encompassing temperature, humidity, wind, hazardous air pollutants, and microbes render stone monuments in outdoor locations more vulnerable to deterioration^9^. Such variable’ cumulative effects, which speed up the process of significant stone monument deterioration and can be categorized as mechanical, chemical, industrial, and biological erosion, rather than their individual effects, make them mainly interconnected. The formation of biofilm on the exterior of stone monuments through biological weathering enhances the weathering process by increasing surface pollution, which results in secondary physical damage^10^. According to Sterflinger and Piar^2^, various kinds of microorganisms like bacteria, fungi, microalgae, and sometimes animals and higher plants trigger the unwanted changes described as the biodeterioration of such essential stones. The mineral and chemical makeup and the physical characteristics of ancient stones, particularly porosity and water absorption, impact how vulnerable they are to microbial deterioration. Interstingly, the heterotrophic creatures known as fungi can resist extreme environmental circumstances. They can take on various forms and release different metabolic chemicals to aid in their survival^11^.

In addition, fungi can weather many materials such as wood, stones, metals, and cement, as well as polymers and various archaeological materials such as mummies, books, and paintings through enzymatic activity and metabolic processes^12^. For the stone monuments exposed to different environmental conditions, fungi can use stone minerals as a nutrient^13^ and secrete various acids that will affect the primary minerals of ancient stones for stone monuments subjected to diverse environmental circumstances^14^. Bio-deterioration control is becoming a global necessity for cultural asset conservation. This study aims to isolate and identify the microorganisms that cause damage to the archaeological stones in the Temple of Hathor in Dendera, Egypt, as well as evaluation of *S. exfoliatus* SAMAH 2021 inhibitory potential against the identified fungal strains, identification of the produced compounds by the selcected strain , determining their toxicological properties and a trial for restoring the archaeological reliefs in the Temple of Hathor in Dandara, Qena, Egypt using the selcected strain products.

## Materials and methods

### Site and sampling

**Figure 1:**
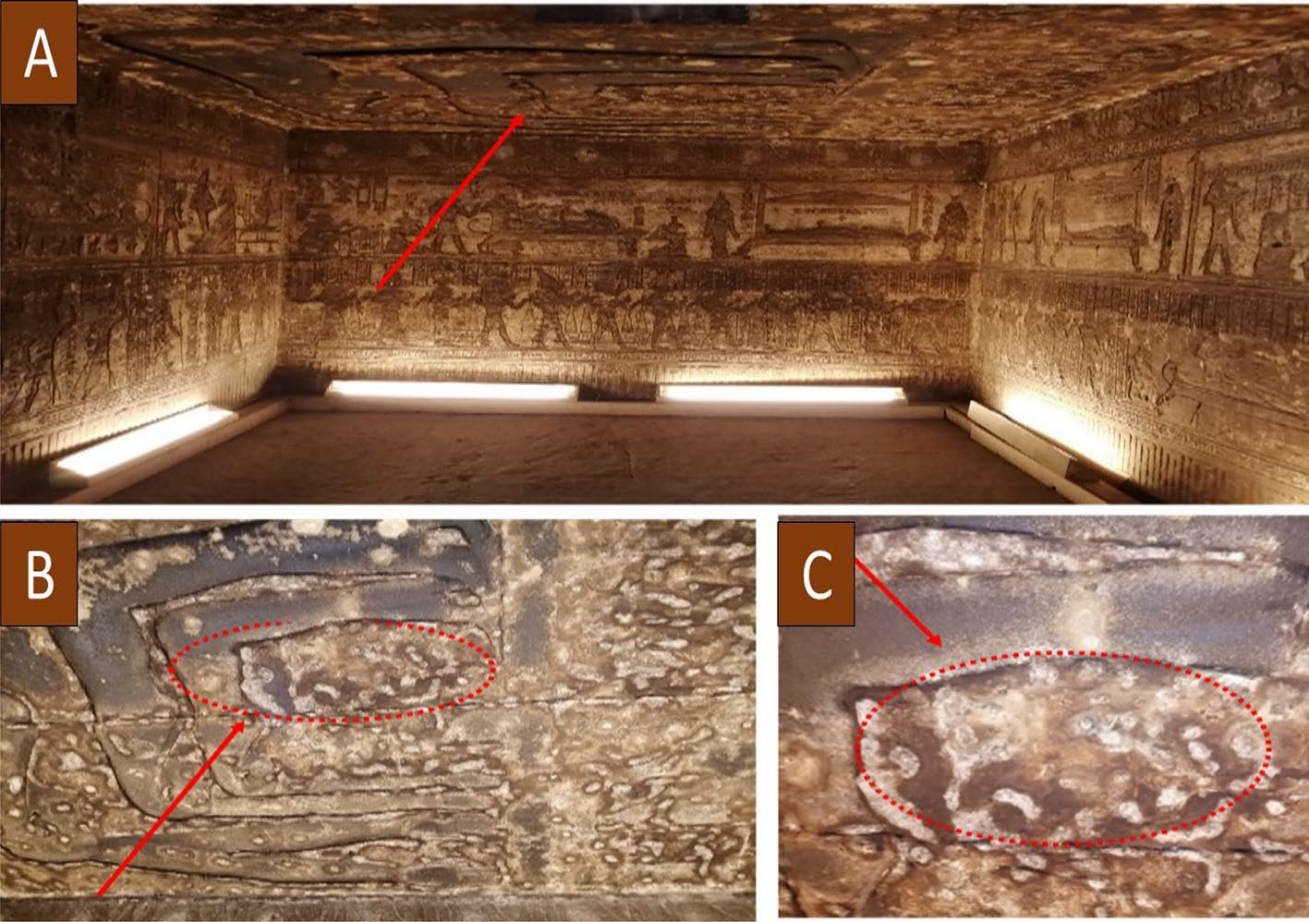
Microbial surface samples at the Temple of Hathor, Luxor, Dendera, Egypt. (**A**, **B**, and **C**) the sampling locations of the wall paintings of an Osirian chapel at Dendera temple. Mahgoub A. Al took this photo by himself in Hathor temple, Luxor, Egypt in August 2022.

The Temple of Hathor at Dendera is located on the west bank of the Nile, roughly 4 km northwest of Qena and 60 km north of Luxor. This temple was dedicated to the goddess Hathor and was constructed between 125 BCE and 60 CE. The temple of Hathor was built of Nubian sandstone quarried in the Aswan province of Upper Egypt. Using sterile cotton swabs, ten microbiological surface samples were obtained from the biofilm established on the surfaces of the degraded bas-reliefs, as illustrated in Fig. 1. All samples were sent to the Microbial Inoculant Center, Faculty of Agriculture, Ain Shams University, Cairo, Egypt for further investigation.

### Microorganisms used in the study

*S. exfoliatus* SAMAH 2021 (OL720220) was obtained from previous work by Abu-Hussien et al.^15^, where it was isolated from soil samples near water effluents in Cairo, Egypt. All pathogenic microbial strains and isolates (*Bacillus cereus* ATCC 11,778, *Escherichia coli* ATCC 8379, *Enterococcus faecalis* ATCC 7080, *Klebsiella pneumoniae* ATCC 00,607, *Pseudomonas aeruginosa* ATCC 27,853, *Salmonella typhi* DSM 17,058, *Shigella sonnei* DSM 5570 and *Staphylococcus aureus* ATCC29737, *Aspergillus flavus* ATCC 9643*, Aspergillus niger* ATCC16404, *Candida albicans* ATCC60193, *Fusarium moniliformin* (isolate), *Fusarium oxysporum* ATCC 62,705, *Rhizoctonia solani* (isolate) were collected from Microbial Resources Center (MIRCEN), Fac. Agric., Ain Shams Univ., Cairo, Egypt. Deteriorative fungi and bacteria were isolated from the Temple of Hathor, Luxor, Dendera, Egypt as described below.

### Media used

All media were obtained from Oxoid, UK, where Casein Starch Medium (CSA) was used to cultivate *S. exfoliatus* SAMAH 2021, it consists of (g/L): Casein Powder 1.0, Strength 10.0, agar 15.0. Oxytetracycline Glucose Yeast Extract Agar (OGYA) was exploited to isolate and preserve the fungal isolates; it consists of (g/L): yeast extract 5.0, glucose 20.0, agar 12.0. Plate count agar (PCA) was used to isolate and maintain bacterial isolates and strains. It consists of (g/L): tryptone 5.0, yeast extract 2.5, glucose 1.0, and agar 15.0. Muller Hinton Agar (MHA) was used to study antibiotic susceptibility testing. All isolates and strains were purified, subcultured at monthly intervals, and maintained at 4 °C for further studies.

### Isolation of the deteriorative fungi and bacteria

Poured plate technique was recommended for counting the selected microbes. For the isolation of fungal and bacterial isolates, OGYA and PCA media were prepared and placed into plates, respectively. All plates were inoculated and incubated for 72 h at 25 °C for fungi and 24 h at 30 °C for bacteria. All colonies were collected and purified using OGYA and PCA subcultures for fungal and bacterial species, respectively, and then stored at 4 °C for further investigations^16^.

### Standard inoculum

Standard inoculum of (1.6–3.8 × 10^8^ CFU/mL) for bacterial isolates and strains was created by inoculating 50 mL of PCB medium in an Erlenmeyer flask (250 mL) with a loopful of tested culture and incubating at 30 °C at 150 rpm for 24 h. Scratching the agar slants in the presence of 10.0 mL of sterilized saline solution yielded spore suspensions for fungal isolates. The spore suspensions (1.1 × 10^8^/mL) collected were utilized as standard inoculum in shake flask tests. Scratched slant cultures of *S. exfoliatus* SAMAH 2021 were resuspended in 50 mL of sterile saline water, and spore concentration was adjusted to 16 × 10^9^ spore/mL^17^.

### Identification of deteriorative bacterial isolates

#### Phenotypic identification

Isolates were identified based on their cultural and cell morphological features. Bacterial isolates were stained using Gram and spore staining. Fungal isolates were grown on slide cultures.

#### Bacterial genotypic identification

Bacterial DNA was extracted, and gene sequencing was applied using molecular based approach using polymerase chain reaction (PCR) to partially amplify the 16S rRNA gene sequence using the two universal primers (F1: 5′ AGAGTTT (G/C) ATCCTGGCTCAG 3′ and R1 5′ ACGG (A/C) TACCTTGTTACGACTT 3′). The partially amplified PCR product was purified using a QIA quick gel extraction kit (Qiagen, Germany). Macrogen company (South Korea) sequenced the 16S rRNA of the purified PCR product. Using BioEdit version 7.0.4, sequence readings were clipped and aggregated, and ClusterW version 4.5.1 was used to align the resulting genomic information. The NCBI database was employed to conduct BLAST inquiries^18^. MEGA 11 software was employed to build phylogenetic trees that use the neighbor-joining cladogram^19^. MEGA software (version 11.0) [Computer software]. Available from https://www.megasoftware.net/.

#### Fungal genotypic identification

For molecular identification (18S rRNA sequencing) and DNA extraction using a Patho-gene-spin DNA/RNA extraction kit provided by Intron Biotechnology Company, Korea, the fungal colonies were transferred to the Molecular Biology Research Unit at Assiut University. Afterward, a fungal DNA sample was shipped to SolGent Company in Daejeon, South Korea, for PCR and rRNA gene sequencing. The PCR of the selected isolates was performed using ITS1 (forward) and ITS4 (reverse) primers as follows: ITS1: (5′-TCCGTAGGTGAACCTGCGG-3′), and ITS4: (5′-TCCTCCGCTTATTGATATGC-3′). The ddNTPs were incorporated into the reaction mixture and the corresponding primers for sequencing the purified recombinant result. Biological antimicrobial activity of *S. exfoliates *metabolites.

The produced standard *S. exfoliatus* SAMAH 2021 inoculum was inoculated into CSB medium at 5% v/v (2 × 10^7^ spores/mL) and incubated at 30 °C for 240 h at 120 rpm. Ten milliliters were taken at 10d intervals. For 15 min, cultures were centrifuged at 10,000 rpm. Pellets were collected to determine cell dry weight (CDW), and the supernatant was collected to determine antibacterial activity as an inhibition zone diameter (IZD) in centimeters (cm). All experiments were done in triplicate. The logarithmic phase regression coefficient was estimated based on the correlation between time (h) and IZD (cm). According to Maier et al.^20^, the specific inhibition rate (d) was calculated as follows:

1 $$ {\text{Specific}}\,{\text{ inhibition}}\,{\text{ rate}}\left( {\upmu _{{\text{d}}} /{\text{h}}} \right) \, = \, \left( {{\text{ln}}\,{\text{ X}}{-}{\text{ln}}\,{\text{ X}}_{0} } \right) \, / \, \left( {{\text{t }} - {\text{ t}}_{0} } \right) $$

where X = IZD after the time (t) and X_0_ = IZD at the beginning time (t_0_).

### Extraction of antimicrobial products

*S. exfoliatus* SAMAH 2021 was inoculated into CSB medium and cultivated for 144 h at 150 rpm at 30 °C. To obtain supernatant, the developed culture was centrifuged at 10,000 rpm for 15 min. As stated by Augustine et al.^21^, the supernatant was collected in test tubes containing ethyl acetate solvent (1:1 (v/v)) and left for 1 h before being separated and evaporated to dryness in an 80–90 °C water bath. CDW (g/L) was determined by collecting pellets.

### Antibiotic susceptibility test

According to CLSI^22^, criteria were followed to identify antibiotic sensitivity in all bacterial and fungal strains using the disc diffusion technique. Oxoid, UK, provided two common commercial antibiotics; Tetracycline (10 µg/mL) and Doxycycline (30 g/mL) used for medical purposes and treatments for bacterial strains. The susceptibility findings were classified as sensitive (S), intermediate (I), and resistant (R). The disc diffusion technique was used for fungal strains with Cefetizime discs (CAZ 30 µg). The IZD was measured in centimeters (cm). *B. cereus*, *E. coli*, *E. faecalis*, *K. pneumoniae*, *P. aeruginosa*, *S. typhi*, *S. sonnei*, and *S. aureus* were the pathogenic clinical bacterial strains. *A. solani*, *A. flavus*, *C. albicans*, and *F. oxysporum* were the fungal strains studied.

### Minimum inhibitory concentration (MIC) of antimicrobial products

At concentrations ranging (from 0, 25, 50, and 100%), *S. exfoliatus* antimicrobial substances (metabolites) were investigated against the four identified bacteria and fungi that had a negative impact on Egyptian antiquities. The wells of Muller Hinton Agar (MHA) plates were loaded with extracted products at serial dilutions of 0, 25, 75, and 100% with a control plate. Individually, 100 µL of each bacterial inoculum and fungal spore solution was dispersed onto the plates’surfaces. The plates were then incubated at 37 °C for bacteria for 24 h and fungi for 3–5 d. IDZ was calaculated in (mm)^23^.

### Colorimetric measurements of *S. exfoliatus* SAMAH 2021 metabolites

For colorimetric alterations, the National Institute of Standards (NIS) in Cairo, Egypt, employed the SDL Company's Optimatch 3100^®^ to measure color change produced by *S. exfoliatus* SAMAH 2021 metabolites on experimental sandstone samples before and after treatment.

The CIE L* a* b* system was used to record the color variations, with the L value equating to brightness, the “a” value to red-green, and the “b” value to yellow-blue. The total color changes (E) before and after treatment were calculated using the following Equation^24^:

2 $$ \Delta E = \sqrt {(\Delta L)^{2} + (\Delta a)^{2} + (\Delta b)^{2} } $$

where L (lightness), a (red/green axis), and b (yellow/blue axis) values were recorded.

### Gas chromatography (GC/MS) analysis for *S. exfoliatus* SAMAH 2021 metabolites

Extracted products of *S. exfoliatus* SAMAH 2021 were dried over anhydrous Na_2_SO_4_ using a rotary evaporator, then dissolved by methanol. A capillary column TG-5MS (30 m × 0.25 mm × 0.25 m film thickness) and a Trace GC-TSQ mass spectrometer (Thermo Scientific, Austin, TX, USA) were used. The column temperature was maintained at 50 °C and raised by 5 °C/min until reaching 250 °C, then maintained for 2 min. expanded by 30 °C/min to an ultimate temperature of 300 °C and held for 2 min. Helium was employed as the carrier gas, with a constant flow rate of 1 mL/min, and temperatures of the injector and MS transfer line were maintained at 270 and 260 °C, respectively. The auto sampler AS1300 paired with the GC in split mode automatically injected diluted samples of 1 μL with a solvent delay of 4 min. EI mass spectra were collected at 70 eV ionization voltages over the range of m/z 50–650 in full scan mode. The ion source temperature was set at 200 °C. The components were identified by comparison of their mass spectra with those of WILEY 09 and NIST 14 mass spectral database^25^.

### Cytotoxicity of *S. exfoliatus* SAMAH 2021 metabolites

Human skin fibroblast (HSF) cells were obtained from Nawah Scientific Inc. (Mokatam, Cairo, Egypt). DMEM media with added antibiotics (streptomycin 100 mg/mL, penicillin 100U/mL, and heat-inactivated fetal bovine serum 10%) were prepared in a CO_2_ humid atmosphere of 5% humidity (v/v) at 37 °C. SRB assay was carried out for the determination of cell viability^26^.

### Statistical analysis

All samples and collected data were statistically analyzed using IBM® SPSS® Statistics software (2017). A Tukey test at a *P*-value of 0.05 was applied.

## Results

### Isolation of the deteriorative microflora

Ten biofilm samples were taken from the degraded bas-relief surfaces of the Hathor temple and inoculated on PCA and OGYA plates for microbial screening. Four isolates were obtained (3 fungal, F1, F2, F3, and one bacterial, B1, isolates). Following successive purification processes, all isolates were identified phenotypically and genotypically.

### Identification of the deteriorative isolates

#### Phenotypic identification

**Figure 2:**
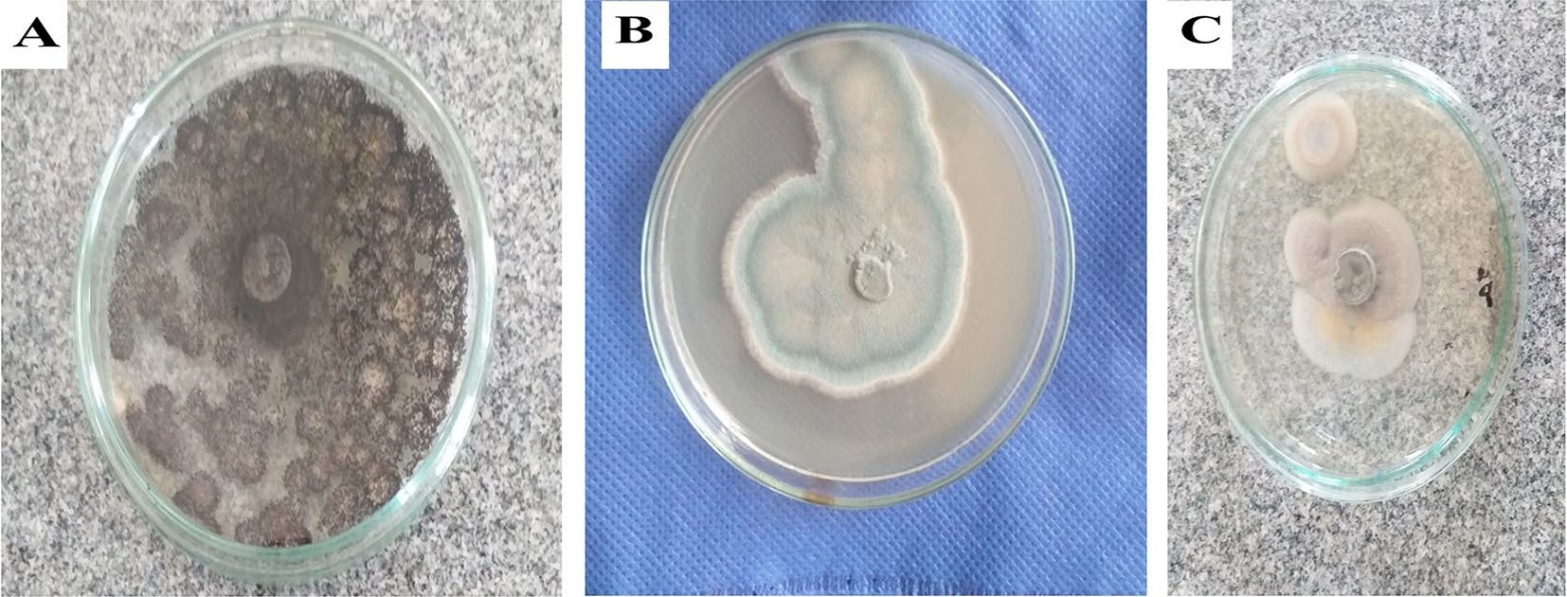
Colonies of the deteriorative fungal isolates. (**a**) F1 fungal isolate, (**b**) F2 fungal isolate. (**c**) F3 fungal isolate.

The bacterial isolate, B1, had a round colony with smooth surface end edges and off-white color. Microscopic examinations confirmed its shape and motility as it had a long rod shape, motile and terminal spores. Fungal isolate F1 had black conidial spores, while F2 isolate exhibited greenish colonies with branching or simple conidiophores in brush-like clusters. Isolate F3 had orange-salmon conidial hypha and dark melanized structures, as shown in Fig. 2.

#### Genotypic identification

**Figure 3:**
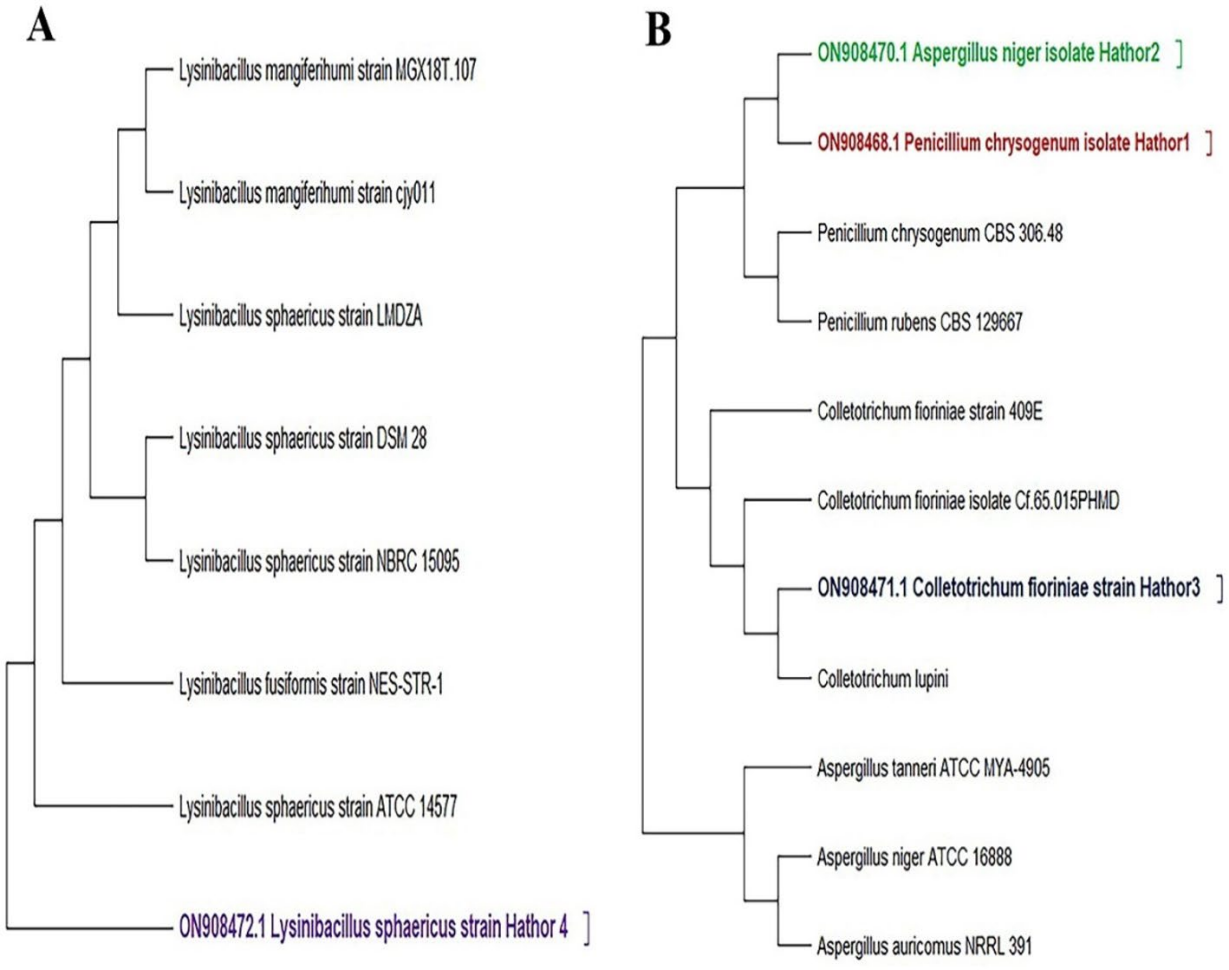
Neighbor-joining trees based on 16S rRNA and 18S rRNA sequences obtained from BLAST search indicating the position of each isolate and related strains. (**a**) Fungal strains. (**b**) bacterial strain.

To recognize the unexplained bacterial and fungal isolates that displayed spoilage organisms' potential for stone monuments, 16S rRNA and 18S rRNA gene sequencing profiling was applied. The phylogenetic trees exhibited here were built together by employing the neighbor-joining algorithm (Fig. 3). Multiple *Bacillus* and *Lysinibacillus* 16S rRNA sequence types were detected in the bacterial phylogenetic tree. The 16S rRNA gene sequence was confirmed to be that of *L. sphaericus* as a consequence of the results depicted in Fig. 3A, showing that it had the accession No. ON908472 recorded in (NCBI, Bethesda, MD, USA). Figure 3, which compares the sequence data for several *Lysinibacillus* isolates, also illustrated the compatibility between the isolates and their immediate phylogenetic neighbors. The *Lysinibacillus* 16S rRNA gene tree included many groups in other sequence categories; however, the phylogenetic branches were generated from a multitude of sequences. The findings revealed an almost 95% sequence similarity between the 10 *Lysinibacillus* spp. and *Lysinibacillus sphaericus*. In the fungal phylogenetic tree, Isolates belonged to *Aspergillus niger* Hathor 2 (accession No. ON908470), *Colletotrichum fioriniae* Hathor 3 (accession No. ON908471), *Penicillium chrysogenum* HATHOR 1 (accession No. ON908468) were depicted in Fig. 3B.

### Biological antimicrobial activity

**Figure 4:**
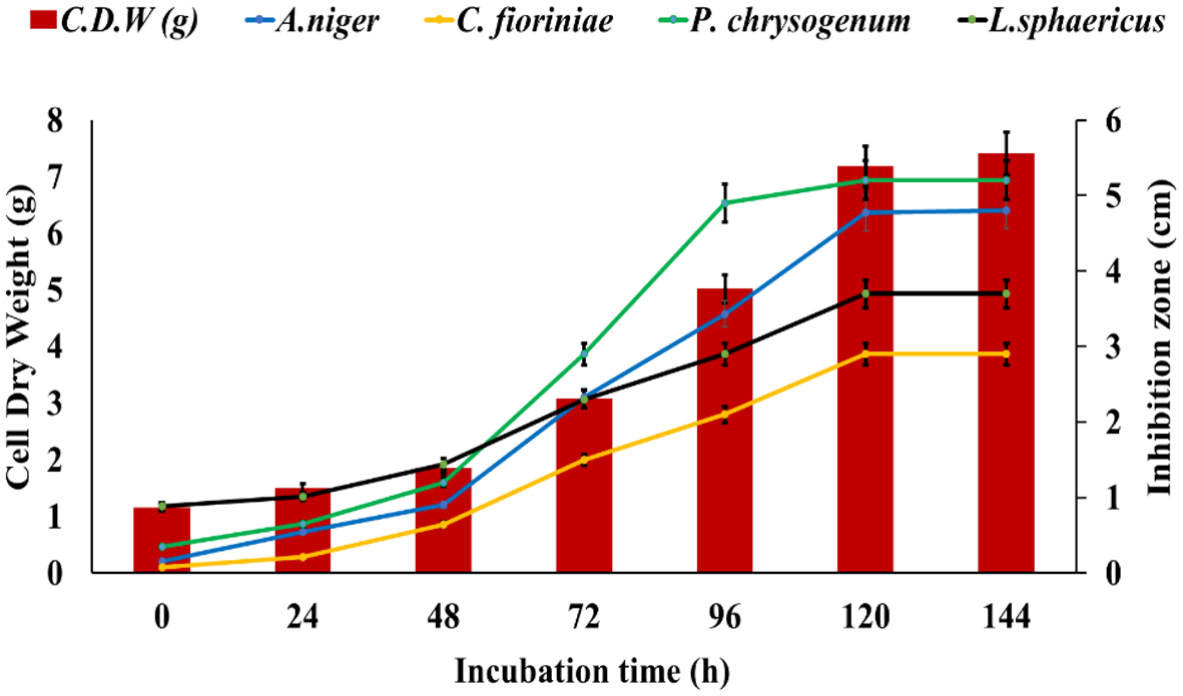
The antimicrobial activity estimation of antimicrobial metabolite (CDW *vis*. IZD) against the bio deteriorative limestone (sandstone) monuments strains.

**Figure 5:**
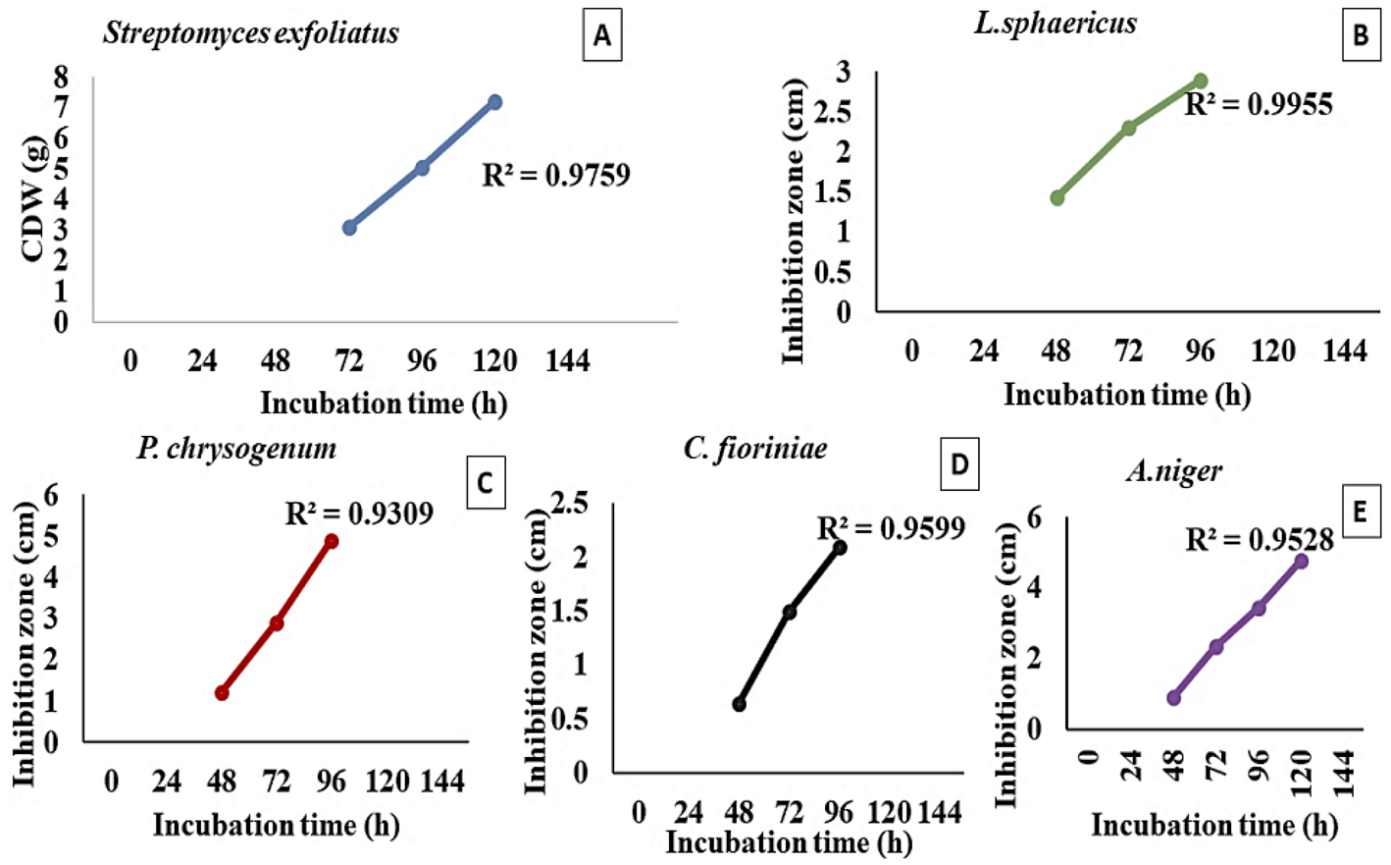
Correlation coefficient (R^2^) between CDW in the logharitmic growth phase of *S. exfoliatus* SAMAH 2021 and IZD of its metabolites against the four obtained strains.

**Figure 6:**
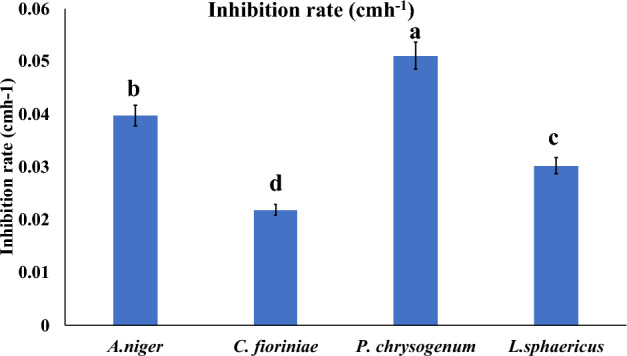
Inhibition rate (cmh^−1^) for the stone monuments deteriorative strains during the logarithmic phase of *S. exfoliatus* SAMAH 2021 antimicrobial.

The antimicrobial activity of *S. exfoliatus* SAMAH 2021 metabolites against the four deteriorative fungal and bacterial strains (*A. niger*, *C. fioriniae*, *P. chrysogenum*, and *L. sphaericus*) was confirmed by calculating the correlation coefficient (R^2^) of *S. exfoliatus* SAMAH 2021 CDW (g/L) against IZD for tested strains. It was recorded that both CDW and IZD achieved their maximum values after 144 h of incubation reached 8 g/L for CDW. All identified strains showed different sensitivity records for the *S. exfoliatus* SAMAH 2021 antimicrobial products. *P. chrysogenum* was inhibited by a zone of 5 cm followed by *A. niger* with an inhibitory zone of 4.8 cm. *L. sphaericus* had intermediate resistant with a zone of 3.8 cm*. fioriniae* was the most resistant strain with a zone of 2.9 cm as illustrated in (Fig. 4). The correlation coefficient of CDW and IZD of *S. exfoliatus* SAMAH 2021 metabolites against identified deteriorative strains reflected a strong correlation ranging between 0.93 and 0.99% for all tested strains (Fig. 5). Growth kinetics for the logarithmic phase revealed that the specific inhibition rate (μ_d_) was 0.05, 0.04, 0.03, and 0.021 cm.h^-1^ for the stone monuments deteriorative strain of *P. chrysogenum*, *A. niger*, *C. fioriniae*, and *L. sphaericus,* respectively, as presented in (Fig. 6).

### Antibiotic and antimicrobial sensitivity for the four obtained deteriorative strains

**Figure 7:**
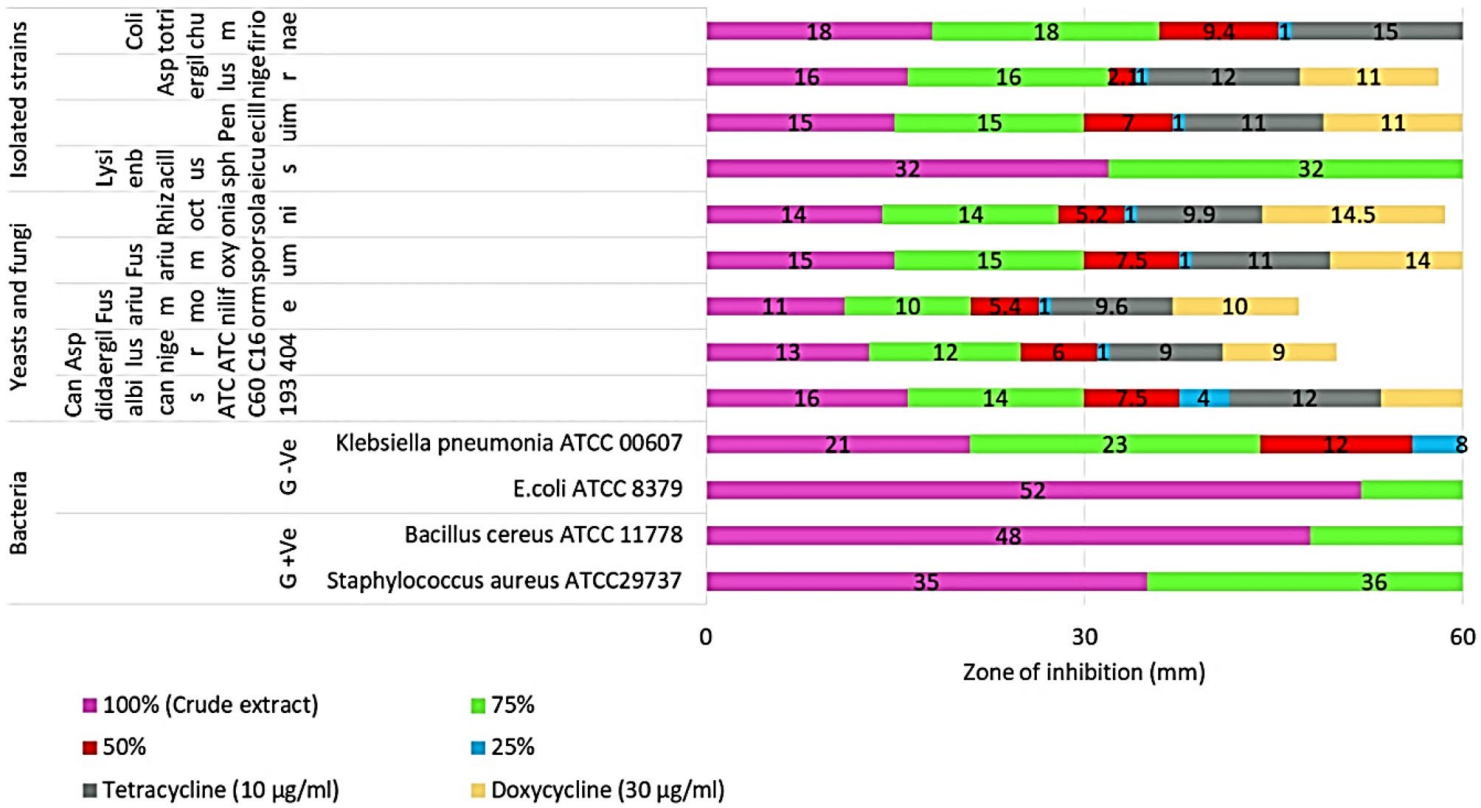
Antimicrobial activity profile of *Streptomyces exfoliatus* SAMAH 2021 antimicrobial products against the four deteriorative strains as well as the pathogenic bacterial and fungal strains on MHA incubated at 30 °C for 24–72 h of incubation, respectively.

**Figure 8:**
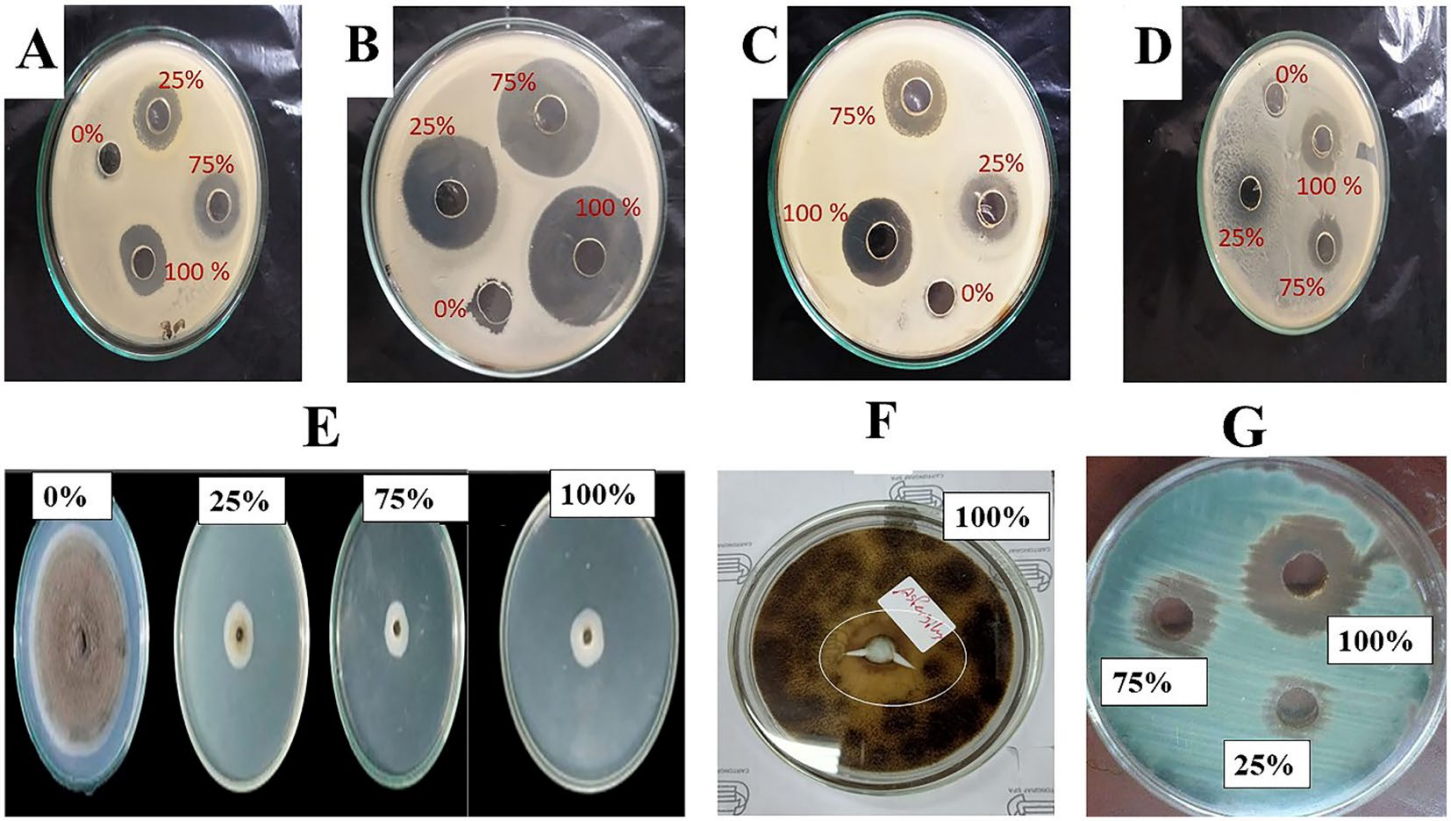
Antimicrobial activity S*. exfoliatus* SAMAH 2021 metabolites at different concentrations (0, 25, 75, 100%) against bacterial strains (**A**–**C**) where, (**A**) *Staphylococcus aureus,* (**B**) *Bacillus cereus*, (**C**) *E.coli* and the four obtained deteriorative strains where, (**D**) *Lysinibacillus sphaericus*, (**E**) growth zone of *Colitotrichum firionae*, (**F**) *Aspergillus niger*, (**G**) *Penicillium chrysogenumon* grown on MHA and incubated at 30 °C for 24 h of incubation.

Table 1 showed that antimicrobial compounds had strong antimicrobial efficacy against pathogenic G^+ve^ and G^−ve^ bacteria, fungi, and yeast when compared to the most widely used commercial antibiotics (tetracycline and doxycycline). At 100% concentration, antimicrobial compounds had an IZD ranging from 2.1 to 5.2 cm, while the commercial antibiotic had an IZD ranging from 0.85 to 4.17 cm. At concentrations ranging from 25 to 75%, *E. coli* had the most significant IZD (5.0–5.2 cm), followed by *B. cereus* (4.7–4.8 cm). *S. aureus* had an IZD of 3.5–3.6 cm, whereas *L. sphaericus* had the lowest IZD of 3.2 cm. Both *A. flavus* and *A. niger* had the smallest IZD, measuring 1.2–1.3 cm and 1.1–1.2 cm, respectively as shown in (Figs. 7, 8). When compared to the prescribed antibiotics, tetracycline and doxycycline, *S. exfoliatus* SAMAH 2021 antimicrobial concentrations of 25 and 50% had a low meaningful impact in all tested isolates.

**Table 1:** Antimicrobial activity and MIC profile of *S. exfoliatus* SAMAH 2021 metabolites against the four deteriorative strains on MHA incubated at 30 °C for 24–72 h of incubation, respectively, in comparison with commercial and standard antibiotics.

Clinical strains			Zone of inhibition (cm)						
			Streptomyces metabolites concentration (%)					Positive control	
			100	75	50	25	0	Tetracycline (10 µg/ml)	Doxycycline (30 µg/ml)
Bacteria	G ^+Ve^	*Staphylococcus aureus* ATCC29737	3.60* ± 0.17^e^	3.50 ± 0.21^ef^*	1.10 ± 0.06^pq^	5.0 ± 0.06^u^	–	2.50 ± 0.03^i^	2.40 ± 0.05^ij^
		*Bacillus cereus ATCC* 11,778	4.80c ± 0.05*	4.70 ± 0.01^cd^*	2.10 ± 0.01^l^	8.0 ± 0.29^st^	–	3.15 ± 0.01^g^*	2.70 ± 0.11^hi^
	G ^−Ve^	*E.coli* ATCC 8379	5.20 ± 0.06^a^*	5.00 ± 0.01^b^*	2.50 ± 0.02^i^	4.0 ± 0.01^v^	–	4.17 ± 0.01^d^*	3.07 ± 0.03^gh^*
		*Klebsiella pneumonia* ATCC 00,607	2.30 ± 0.01^jk^	2.10 ± 0.01^l^	1.20 ± 0.06^p^	8.0 ± 0.08^rs^	–	1.80 ± 0.01^m^	1.80 ± 0.01^m^
Isolated strains		*Lysinibacillus sphaericus strain* EGYSTONE	3.20 ± 0.01f.*	3.20 ± 0.02f.*	1.75 ± 0.08^m^	5.0 ± 0.01^u^	–	2.10 ± 0.01^l^	2.00 ± 0.02^lm^
								Cefetizime discs (CAZ 30 µg)	
Yeasts and fungi	*Candida albicans* ATCC60193		1.60 ± 0.05^mn^	1.40 ± 0.01^o^	0.75 ± 0.01^st^	0.40 ± 0.01^v^	–	1.20 ± 0.02^p^	1.50 ± 0.01^n^
	*Aspergillus niger* ATCC16404		1.30 ± 0.01^op^	1.20 ± 0.02^p^	0.60 ± 0.01^t^	0.10 ± 0.01^w^	–	0.90 ± 0.02^r^	0.90 ± 0.01^r^
	*Aspergillus flavus* ATCC 9643		1.20 ± 0.11^p^	1.10 ± 0.01^pq^	0.54 ± 0.01^tu^	0.10 ± 0.03^w^	–	0.85 ± 0.05^rs^	0.90 ± 0.01^r^
	*Fusarium moniliformin* (isolate)		1.10 ± 0.05^pq^	1.00 ± 0.01^q^	0.54 ± 0.01^tu^	0.10 ± 0.02^w^	–	0.96 ± 0.02^qr^	1.00 ± 0.01^q^
	*Fusarium oxysporum* ATCC 62,705		1.50 ± 0.17^n^	1.50 ± 0.01^n^	0.75 ± 0.01^st^	0.10 ± 0.06^w^	–	1.10 ± 0.04^pq^	1.40 ± 0.01^o^
	*Rhizoctonia solani* (isolate)		1.40 ± 0.17^o^	1.40 ± 0.01^o^	0.52 ± 0.01^u^	0.10 ± 0.03^w^	–	0.99 ± 0.05^q^	1.45 ± 0.01^no^
Isolated strains	*Penecilluim chrysogenum* strain EGYSTONE1 2022		1.50 ± 0.04^n^	1.50 ± 0.01^n^	0.70 ± 0.10^st^	1.0 ± 0.01^w^	–	1.10 ± 0.03^pq^	1.10 ± 0.01^pq^
	*Aspergillus niger* strain EGYSTONE2 2022		1.60 ± 0.15^mn^	1.60 ± 0.01^mn^	0.21 ± 0.02^vw^	1.0 ± 0.51^w^	–	1.20 ± 0.01^p^	1.10 ± 0.07^pq^
	*Colitotrichum firionae* strain EGYSTONE1 2022		1.80 ± 0.04^m^	1.80 ± 0.02^m^	0.94 ± 0.01^r^	1.0 ± 0.06^w^	–	1.50 ± 0.13^n^	1.20 ± 0.01^l^

Values are mean averages of three independent assays ± standard deviations. Values by * (*P* ≤ 0.05) are significant means, according to the Tukey’s test at a 5% level.

Different letters indicate significant differences, according to Tukey’s Studentized Range (HSD) Test (*p* < 0.05).

### Colorimetric measurements of *Streptomyces exfoliatus* SAMAH 2021 metabolites

**Figure 9:**
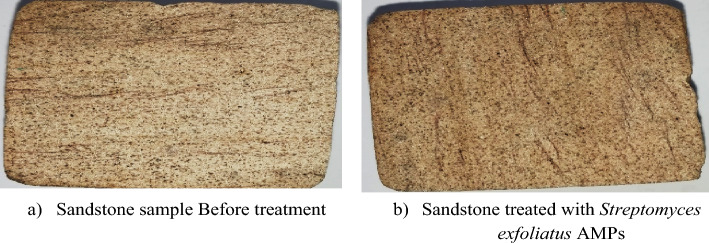
Color changes of experimental sandstone samples before and after treatment with *Streptomyces exfoliatus* SAMAH 2021 metabolites.

The total color changes (ΔE) of samples before/after treatment indicated a change in the chromaticity values. The experimental sandstone sample treated with *Streptomyces exfoliatus* SAMAH 2021 metabolites don’t show any significant change in comparison with the control sandstone sample (ΔE = 0.1) as shown in Table 2 and Fig. 9. Based on previous results, the use of *Streptomyces exfoliatus* SAMAH 2021 metabolites does not cause any significant change in color values in contrast with conservation ethics.

**Table 2:** Color changes of experimental sandstone samples before and after treatment with *Streptomyces exfoliatus* SAMAH 2021 metabolites.

	Before treatment (control)	After treatment	Color changes	
L	73 ± 1^a^	73 ± 1.73^a^	Δ L	0.0
a	5 ± 1^c^	5 ± 1^c^	Δ a	0.0
B	16 ± 1^b^	17 ± 1^b^	Δ B	0.1
			Δ E	0.1

Values are mean averages of three independent assays ± standard deviations. Values by * (*P* ≤ 0.05) are significant means, according to the Tukey’s test at a 5% level.

Different letters indicate significant differences, according to Tukey’s Studentized Range (HSD) Test (*p* < 0.05).

### GC–MS analysis of antimicrobial agents from *S. exfoliatus* SAMAH 2021

**Figure 10:**
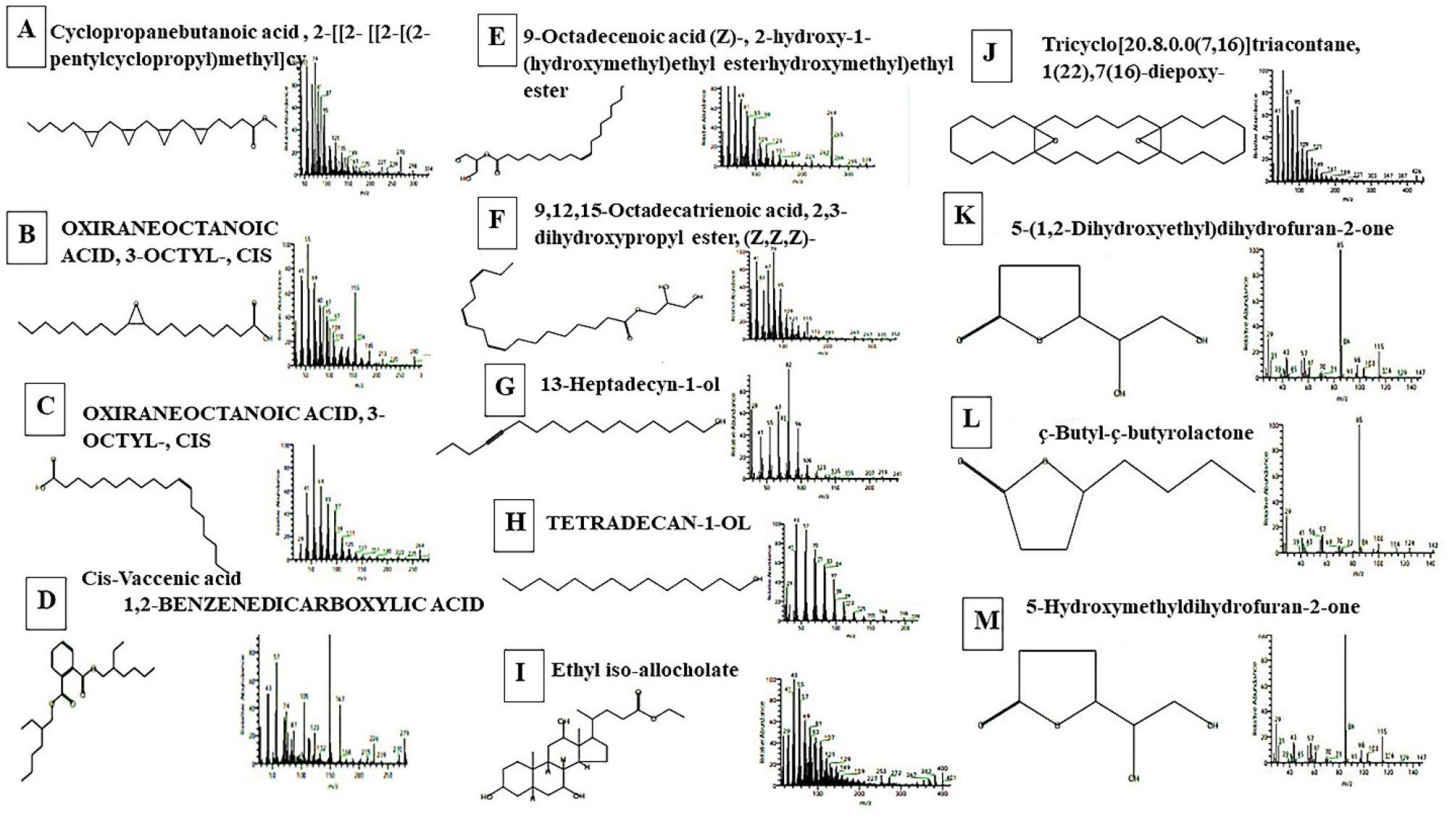
GC–MS Chromatograms of antibiotic agents identified in the methanolic extract of *S. exfoliatus* SAMAH 2021.

GC–MS analysis is used to determine the production of novel antibiotics by *S. exfoliates*. Thirteen bioactive compounds were identified in the methanolic extract of S. *exfoliatus* SAMAH 2021 (Cyclopropane butanoic acid, 2- [[2- [[2- [(2-pentylcyclopropyl)meth yl]cyclopropyl]methyl]cyclopropyl] methyl]-, methyl ester (antimalarial, antituberculosis and antifungal); Oxiraneoctanoic acid,3-octyl-, cis Cis-vaccenic acid; 1,2-Benzene dicarboxylic acid; 9-Octadecenoic acid (Z)-, 2-hydroxy-1-(hydroxymethyl)ethyl ester hydroxymethyl)ethyl ester; 9,12,15-Octadecatrienoic acid, 2,3-dihydroxy propyl ester, (Z, Z, Z)-; Tetradecan-1-ol; 13-Heptadecyn-1-ol; Ethyl iso-allo cholate; Tricyclo[20.8.0.0(7,16)]triacontane, 1(22),7(16)-diepoxy-; 5-(1,2-Dihydroxy ethyl)dihydrofuran-2-one; ç-Butyl-ç-butyrolactone and 5-Hydroxymethyldihydrofuran-2-one as illustrated in Table 3 and Fig. 10.

**Table 3:** Major Antibiotic compounds identified in the methanolic extract of *S. exfoliatus* SAMAH 2021.

No	Antibiotic compounds	RT (min)	Area%	Molecular weight	Molecular formula
1	Cyclopropane butanoic acid , 2-[[2- [[2-[(2-pentylcyclopropyl)methyl]cy	35.64	3.73	374	C_25_H_42_O_2_
2	OXIRANEOCTANOIC ACID, 3-OCTYL-, CIS	33.34	0.52	298	C_18_H_34_O_3_
3	cis-Vaccenic acid	30.25	4.73	282	C_18_H_34_O_2_
4	1,2-BENZENEDICARBOXYLIC ACID	36.61	2.08	390	C_24_H_38_O_4_
5	9-Octadecenoic acid (Z)-, 2-hydroxy-1-(hydroxymethyl)ethyl ester hydroxymethyl)ethyl ester	38.38	0.76	356	C_21_H_40_O_4_
6	9,12,15-Octadecatrienoic acid, 2,3-dihydroxypropyl ester, (Z,Z,Z)-	35.64	3.73	352	C_21_H_36_O_4_
7	TETRADECAN-1-OL	4.15	4.70	214	C_14_H_30_O
8	13-Heptadecyn-1-ol	30.86	0.41	252	C_17_H_32_O
9	Ethyl iso-allo cholate				
10	Tricyclo[20.8.0.0(7,16)]triacontane, 1(22),7(16)-diepoxy-	43.37	1.54	444	C_30_H_52_O_2_
11	5-(1,2-Dihydroxy ethyl)dihydrofuran-2-one	6.29	7.48	146	C_6_H_10_O_4_
12	ç-Butyl-ç-butyrolactone	6.29	7.48	142	C_8_H_14_O_2_
13	5-Hydroxymethyldihydrofuran-2-one	6.29	7.48	116	C_5_H_8_O_3_

### Cytotoxicity

**Figure 11:**
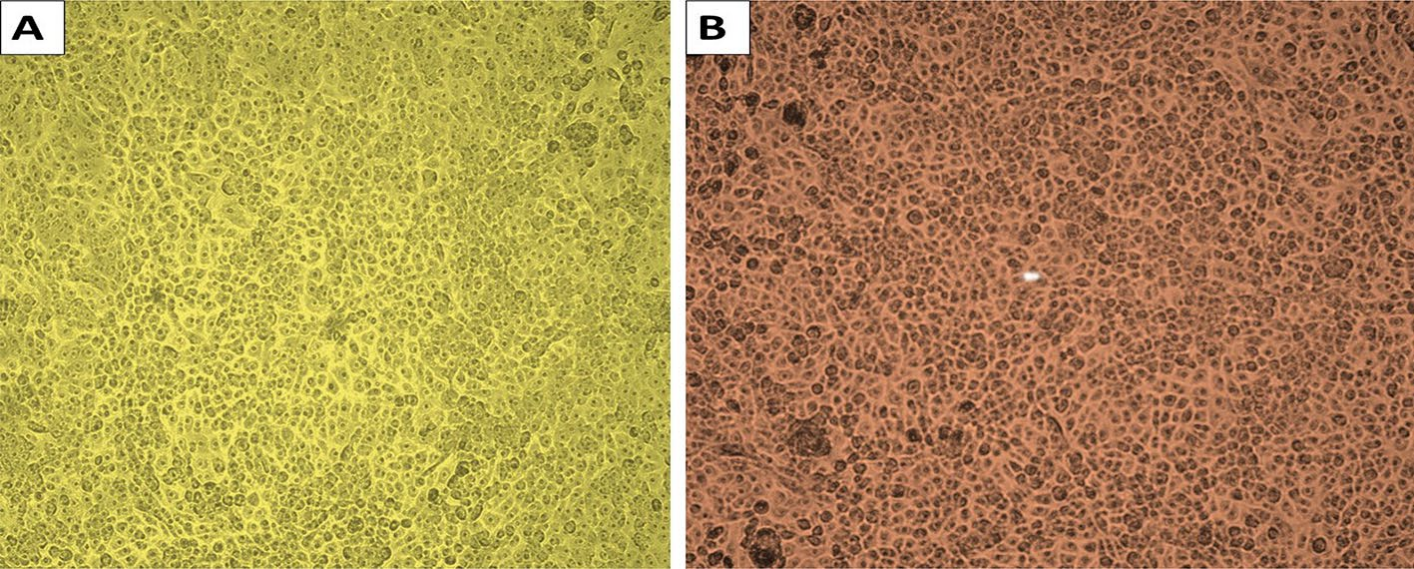
Cytotoxicity of the *S. exfoliatus* SAMAH 2021 antibacterial on healthy HSF cells maintained in DMEM media with 100 mg/mL streptomycin, 100U/mL penicillin, and 10% of heat-inactivated fetal bovine serum in humidified 5% (v/v) CO_2_ atmosphere warmed at 37 °C. Healthy adherent cells are depicted in (**A**) untreated control. (**B**) The existence of regular adhering cells is proven by the antibacterial agent from *S. exfoliatus* SAMAH 2021 being 100% cytotoxic.

Using an MTT assay, four different concentrations of the active metabolites from *S. exfoliatus* SAMAH 2021 (0–100%) were examined towards standard HSF cells. Figure 11 showed no adverse consequences at all doses of the antimicrobial products in functional HSF cells. Cell viability increased to 100%, where it was 97%. 100% of HSF cells were viable under the control condition. We calculated the half-maximal inhibitory concentration (IC50), which was > 100%, using GraphPad Prism (5). When compared to the control treatment, microscopic pictures for *S. exfoliatus* SAMAH 2021 antimicrobial agents exhibited no cytotoxicity.

## Discussion

The microbial deterioration of Egyptian stone monuments emphasizes the need for finding efficient solutions to mitigate the effects of biodeterioration and enhance the international consciousness about the biopreservation of our historical heritage. Studies should focus on discovering new alternatives for the chemical antimicrobial compounds which had harmful effects on man and environment as well^27^. Our results revealed that 75% of the collected deteriorative isolates were fungi rather than bacteria indicating the gross contamination of museums, temples, and tombs. This fungal contamination is due to the low temperatures and high relative humidity to reach 70% in indoors that encourages fungal growth by germinating and spreading their spores especially xerophilic and xerotolerant species such as *Aspergillus sp., Penecillium* sp., and *Wallemia* sp.^28^.

Dessimentaion of fungal strains in museums is also influenced by the presence of air borne minerals, carbonates, and other combounds^29^. There are two types of deteriorative fungi, the Epileptic fungi (living on the rock) and endolithic fungi (living inside pores and fissures)^30^ such as such as *Penicillium frequentens, and Aspergillus fumigatus*. Black fungi (*Aspergillus* sp.) is responsible for stone monuments biodeterioration. Likewise, it has been found that *Penicillium* sp. was related to the formation of dark biofilms on the outer layer of landmarks^31^. *L. sphaericus* is a harmful bacterium forms reistant endospores to high temperatures, synthetic compounds, and intense light causing biofilm formation on stone surfaces^32^. Our findings were consistent with Salvadori and Municchia^33^, who recovered *A. niger* and *Penicillium* sp. from stone monuments. Also, *P. frequentens* and *Cladosporium cladosporoides* were isolated by Mohammadi et al.^34^ from stone monuments. It was also reported that *A. niger* was the most abundant species found on limestone, marble, and sandstone. In addition to previous,* A. flavus* and *Fusarium roseum* were found on land sites, limestone, marble, and sandstone^35^**.**

*Streptomyces* sp., is well known as the greatest producer for secondary metabolites especially antimicrobial products^36^. In the current study, numerous bioactive substances that act as antimicrobial agents were identified by (GC–MS) analysis. Ethyl iso-allocholate; 13-heptadecyn-1-ol and 9,12,15-Octadecatrienoic acid 2,3-dihydroxy propyl ester, (Z, Z, Z) possess antimicrobial activity (antiviral, anti-bacterial, and anti-fungal)^37^. The primary metabolites of *S. exfoliatus* SAMAH 2021 are fatty acids and fatty acid ester. Generally, they were more abundant in *S. exfoliatus* SAMAH 2021 growth cultures such as Cis-Vaccenic acid; 9-Octadecenoic acid (Z)-,2- hydroxy-1-(hydroxymethyl) ethyl ester and Oxiraneoctanoic acid, 3-octyl-, methyl ester.

*Streptomyces* has reported to produce peptides/glycopeptides, angucyclinone, tetracyclines, phenazine, macrolide, anthraquinone, polyene, nonpolyene, benzoxazolophenanthridine, heptadecaglycoside, lactones, and other antibiotics^38^. Many antibiotic compounds have been identified from *Streptomyces fradiae*, including 13 Heptatriacotanol; Cyclopropane butanoic acid, 2-[[2- [[2-[(2-pentylcyclopropyl) methyl]cy; Cis-Vaccenic acid; 9-Octadecenoic acid (Z)-,2- hydroxy-1-(hydroxymethyl)ethyl ester and Oxiraneoctanoic acid, 3-octyl-, methyl ester^39^. It is well known that unsaturated fatty acids have high antimicrobial activity rather saturated fats due to the presence of double bonds^40^. Moreover, free fatty acid surfactants have a high potential to damage cell membranes' stability as well as the inhibition of electron transport chain-related enzymes^41^. Tetradecan-1-OL is one of the found bioactive compounds. It likes fatty alcohol and could be used for antibacterial and antifungal purposes. Also, 1, 2-benzene dicarboxylic acid has antibacterial properties. Chemically, ç-Butyl-ç-butyrolactone and 5-Hydroxymethyldihydrofuran-2-one are described as intramolecular esters of hydroxycarboxylic acids with variable cyclic diameters^42^. Lactones are cytotoxic, antiviral, and antibacterial agents that inhibit microbial growth^43^.

The color variations of experimental sandstone samples before and after treatment with *S. exfoliatus* SAMAH 2021 metabolites indicate the reflection spectra of materials damaged by the obtained strains of *C. fioriniae* strain Hathor 3, *P. chrysogenum* strain HATHOR 1, and *L. sphaericus* strain Hathor 4. The color difference (L) values, the rate of the darkness(%) and color difference (ΔE) for the deteriorating stone samples revealing that the limestone didn’t affect by *S. exfoliatus* SAMAH 2021 metabolites.

Our results emphasized that metabolites produced by *S. exfoliatus* SAMAH 2021 are safe to human and could be used as a biocontrol agent against the most deteriorative fungi and bacteria for Egyptian stone monuments. However, more further studies are indeed needed to ensure the safety concerns of metabolite’s treatment on the monuments along time.

## Conclusion

In the current study, three deteriorative fungi and one bacterial strain (*A. niger*, *C. fioriniae*, *P. chrysogenum*, and *L. sphaericus)* that showed deteriorative activity on the Egyptian stone monuments were picked up and isolated from the Temple of Hathor. Applying *S. exfoliatus* SAMAH 2021 antimicrobial products was an effective method for protecting and preserving our Egyptian stone monuments as it had an inhibitory effect toward all tested deteriorative pathogens with a minimum inhibition concentration (MIC) of 25%. GC–MS analysis showed the existence of 13 compounds in the *S. exfoliatus* SAMAH 2021 products. The light change studies indicated no color or surface change for the limestone piece treated with *S. exfoliatus* SAMAH 2021 antimicrobial products as well as the safe usage of such antimicrobial products as proved by the toxicity experiment.

## Data Availability

The datasets generated during the current study are available in the [NCBI] repository, [https://www.ncbi.nlm.nih.gov/nuccore/OL720220]. https://www.ncbi.nlm.nih.gov/nuccore/ON908471https://www.ncbi.nlm.nih.gov/nuccore/ON908472https://www.ncbi.nlm.nih.gov/nuccore/ON908470https://www.ncbi.nlm.nih.gov/nuccore/ON908468.
